# Radiographic examination of keel bone damage in living laying hens of different strains kept in two housing systems

**DOI:** 10.1371/journal.pone.0194974

**Published:** 2018-05-09

**Authors:** Beryl Katharina Eusemann, Ulrich Baulain, Lars Schrader, Christa Thöne-Reineke, Antonia Patt, Stefanie Petow

**Affiliations:** 1 Institute of Animal Welfare and Animal Husbandry, Friedrich-Loeffler-Institut, Celle, Germany; 2 Institute of Farm Animal Genetics, Friedrich-Loeffler-Institut, Mariensee, Germany; 3 Institute of Animal Welfare, Animal Behavior and Laboratory Animal Science, Freie Universität Berlin, Berlin, Germany; Gaziosmanpasa University, TURKEY

## Abstract

A high prevalence of deviations and fractures of the keel bone is a widespread welfare problem in laying hens. The aim of this study was to experimentally investigate this multifactorial problem throughout the laying period and to compare the prevalence and severity in different layer lines and different housing systems. High performing white (WLA) and brown (BLA) pure bred layer lines and low performing white (R11, G11) and brown layer lines (L68) were kept in both single cages and a floor housing system. A total of 97 hens (19 or 20 from each line, respectively) were repeatedly radiographed in the 35^th^, 51^st^ and 72^nd^ week of age. Fracture prevalence increased with age (p<0.001). The proportion of deviated keel bone area increased only for caged BLA, WLA and R11 hens (p<0.05) and was significantly higher for caged WLA and R11 hens compared to floor-housed WLA and R11 hens in the 72^nd^ week of age (p<0.05). In the 72^nd^ week of age hens in the floor housing system showed significantly more fractures than hens kept in cages (p<0.05). Prevalence of keel bone deviations was significantly higher in the white layer line R11 but significantly lower in the white layer line G11 compared to both brown layer lines and WLA (p<0.05). Brown layers showed significantly more fractures than white layers (p<0.05) in the 51^st^ and 72^nd^ week of age. Within the brown layers there was a significantly lower prevalence of deviations (p<0.05) and fractures (p<0.05) in the low performing (L68) compared to the high performing line (BLA). Our results show a different development of keel bone damage in caged compared to floor-housed hens under experimental conditions. Additionally, they indicate genetic effects on keel bone damage.

## Introduction

Keel bone fractures are one of the most serious animal welfare problems in the egg production industry [1–3]. The prevalence is very high: About 5.5% of hens at the onset of lay and up to 97% of the hens near the end of a production cycle are affected [4–8]. In several studies it has been shown that affected animals are likely to suffer pain [9, 10]. Beside broken keel bones there is also a high prevalence of deviated keel bones. These have been described by Casey-Trott et al. [11] as “bone[s] with an abnormally shaped structure that has not resulted from a fracture but contains section(s) that vary from a theoretically perfect 2-dimensional straight plane in either the transverse or sagittal planes. Additionally, indentations along the ventral surface can also be classified as a deviation.” Reported percentages of keel bone deviations range from 2.6% to 82% [12, 13].

In addition to being a welfare problem, keel bone damage (KBD), i.e. deviations and fractures, might also be an economical problem due to reduced egg production, reduced egg weight and higher feed and water intake [14–16].

The etiology of both fractures and deviations is not yet clear but the causes seem to be numerous. One major factor seems to be the housing system. Wilkins et al. [7] found the lowest prevalence of keel bone fractures in furnished cages compared to all other investigated systems (free-range, organic static and barn), except organic mobile. Petrik et al. [4] found a higher keel bone fracture prevalence in floor-housed compared to cage housed flocks and Keutgen et al. [17] reported a higher prevalence of deviations in free-range and floor housing than in conventional cages. Several authors explain these findings by a higher risk of accidents and collisions in more extensive systems [7, 18] and by the presence of perches in alternative systems and furnished cages but not in conventional cages [18–20]. This explanation is supported by findings that shape and material of perches seem to have an influence on KBD. Käppeli et al. [21] found more deviations in pens with metal perches compared to pens with plastic perches. Stratmann et al. [22] were able to reduce the prevalence of fractures and deviations by covering metal perches with a soft cushion. In terms of fractures, the authors supposed that the compressible cushion absorbed part of the kinetic energy during collisions resulting in reduced energy transferred to the keel bone. In terms of deviations, their results can be explained by findings of a study by Pickel et al. [23] in which peak forces and contact area between perch and keel bone were measured. The authors found a lower peak force and larger contact area in perches with a soft cushion.

However, perches and the higher amount of movement in more extensive systems have also been shown to positively influence breaking strength and mineralization of long bones [24–27]. Movement, such as wing flapping, flying and load-bearing movements on perches and other structures, is important to preserve structural bone [27] which explains these findings.

The phylogenetic background of laying hens seems to affect the susceptibility of keel bones to fractures and deviations. Vits et al. [19] found more keel bone deviations in Lohmann Brown (LB) compared to Lohmann Selected Leghorn (LSL) hens. This finding was confirmed by Habig et al. [28] who added that keel bone deviations of LB hens were also more severe than those of LSL hens. In a study by Wahlström et al. [29] LSL hens were less susceptible to keel bone deviations when compared to a cross-breed of Leghorn and Rhode Island Red-line, called SLU-1329. However, Heerkens et al. [5] found a higher prevalence of keel bone deviations in Dekalb White hens compared to ISA Brown hens. The fracture incidence, in contrast, was higher for the ISA Brown hens. A detailed study about the influence of genetics on the prevalence of keel bone fractures has recently been published by Candelotto et al. [30]. The authors analyzed the susceptibility to keel bone fractures of different crossbred and pure lines with an impact test apparatus that minimized behavioral confounds. They found a reduced likelihood of experimental fractures in the crossbred line Experimental Brown (EB) compared to all other lines and a reduced likelihood of experimental fractures in the commercial line Dual Brown (DB) compared to the commercial line Dekalb White (DW).

Moreover, laying performance is likely to affect prevalence of keel bone damage as selection on high performance has led to a high demand for calcium to produce the egg shell. This might affect bone characteristics, since part of the calcium is mobilized from the bone, especially the medullary bone [31, 32]. However, little is known about the influence of laying performance on keel bone health. Hocking et al. [33] compared different commercial layer lines which showed a high laying performance to traditional lines which showed a lower laying performance. The authors found a significantly higher radiographic density of keel bones and tibiotarsi of traditional compared to commercial layer lines. Moreover, breaking strength of humeri and tibiotarsi was also higher in traditional than in commercial breeds while there was no difference in egg shell strength. The authors concluded that “eggshell quality is maintained in genetically selected lines at the expense of bone strength and bone radiographic density”.

In order to better understand the etiology of KBD it is crucial to consider its development throughout the laying period. Several studies found an increasing prevalence of deviations and fractures with increasing age [5, 21, 26, 28, 29]. However, in some studies, the prevalence of KBD remained constant or even decreased after about the 50^th^ week of age [4, 22]. The method which is mostly used to assess KBD throughout the laying period is the palpation of the keel bone [4, 5, 21, 22, 28]. An alternative method which would give more detailed information about the health status of the keel bone and allow conducting longitudinal studies is radiography. However, although a few studies have shown that it is a reliable tool to detect keel bone fractures [34–36] and deviations [35], radiography has not been used to assess keel bone damage throughout the laying period so far.

The aim of this study was to get a better insight into the influence of housing system, layer line and age on keel bone fractures and deviations within the same experimental design. Therefore, we established an experimental setup with five pure bred layer lines differing in their phylogenetic background (brown and white layer lines) and laying performance (high and low performing lines). Hens of all lines were kept in two different housing systems (single cages and floor group housing) within the same facility so that confounding factors such as different feed and management in the housing systems were minimized. To be able to investigate keel bone fractures and deviations throughout the laying period we took radiographs of all laying hens at three different weeks of age. With this method we also aimed to clearly differentiate between fractures and deviations and to provide an objective measure of the dimension, i.e. severity of deviations.

In view of findings of other studies we predicted that brown purebred lines would show more deviations and fractures compared to white purebred lines and that the high performing lines would show more keel bone damage than the low performing lines. We also predicted that prevalence of deviations would be higher in hens kept in cages enriched with perches compared to hens in the floor housing system and that deviations would be more severe in caged hens. In contrast, we hypothesized that hens in the floor housing system would show more fractures compared to hens kept in single cages. Lastly, we predicted that keel bone damage would increase with age.

## Animals, materials and methods

### Birds and housing conditions

This experiment was performed in accordance with the German Animal Protection Law and approved by the Lower Saxony State Office for Consumer Protection and Food Safety (No. 33.9-42502-05-10A079).

We examined five different pure bred lines of laying hens (*Gallus gallus domesticus*): three closely related white layer lines (WLA, R11, G11) and two closely related brown layer lines (BLA, L68). The two lines WLA and BLA (Lohmann Tierzucht GmbH, Cuxhaven, Germany) have been selected for laying performance and lay around 320 eggs per year. The other lines, R11, G11 and L68 (Friedrich-Loeffler-Institut, Institute of Farm Animal Genetics, Mariensee, Germany) are low performing lines with an average laying performance of 200 eggs per year (see also [37]). Laying maturity, defined as age at the first egg laid, was reached in the 20^th^ week of age in WLA and BLA and in the 24^th^ week of age in R11, G11 and L68 (see also [37]). Moreover, the layer lines differed in body mass: Both brown layer lines were heavier than the three white layer lines (mean body weight and standard deviation in the 72^nd^ week of age: BLA: 1769 ± 205 g, L68: 2036 ± 212 g, WLA: 1564 ± 148 g, R11: 1440 ± 150 g, G11: 1456 ± 160 g).

All chicks (BLA: n = 162, L68: n = 163, WLA: n = 166, R11: n = 159, G11: n = 207) were hatched on the same day and the animals were raised in a floor housing system until 16 weeks of age. Birds of the different layer lines were kept in separate rearing compartments of 24 m^2^ each that were littered with wood-shavings and straw. Perches were provided in the form of two wooden ladders each consisting of five rungs. Each rung was 92 cm in length and squared. The height of each rung was 5 cm and the width 4 cm. The distance between two rungs measured 40 cm and each ladder was 200 cm long. The ladders were leaned against the wall so that the highest rung was at a height of 150 cm. A standard light program was applied throughout the rearing period and a conventional complete feed for chicks (until 7 weeks of age; 12.97 MJ AMEn/kg DM, 189.61 g/kg crude protein, 31.38 g/kg crude fat, 9.14 g/kg Ca, 6.94 g/kg P) and pullets (from 8 to 16 weeks of age; 12.82 MJ AMEn/kg DM, 151.67 g/kg crude protein, 30.21 g/kg crude fat, 15.83 g/kg Ca, 8.11 g/kg P) as well as water were offered *ad libitum*.

At 16 weeks of age, a subset of the hens (BLA: n = 48, L68: n = 48, WLA: n = 49, R11: n = 48, G11: n = 47) was moved and housed individually in single cages (50 cm × 46 cm × 43 cm) for the remainder of the experiment. Each cage was equipped with a food trough, two drinking nipples and a perch. Siblings of the caged birds (n = 30 for each layer line) were moved to floor housing compartments and kept in groups of 15 hens of the same line, resulting in two compartments per line. All compartments measured 2 m x 2 m, were littered with wood-shavings and provided with perches and nests mounted on an elevated slatted floor 0.5 m above the littered area.

In both housing systems the duration of the light period increased gradually from 9 h (16^th^ week of age) to 14 h light (20^th^ week of age). All laying hens were fed *ad libitum* on a conventional laying hen diet (11.68 MJ AMEn/kg DM, 168.11 g/kg crude protein, 29.43 g/kg crude fat, 50.05 g/kg Ca, 5.06 g/kg P) and had *ad libitum* access to water.

### X-ray examinations

Ten hens of each layer line and housing system were selected for X-ray examinations, resulting in 20 hens per layer line. From the lines WLA, R11 and G11 in the floor housing system only nine birds were X-rayed. This was due to a genetic study which was linked to the current study and which required the examination of one offspring each of ten different roosters. Due to losses in the layer lines WLA, R11 and G11 before the start of the experiment, offspring of only nine roosters were available in the floor housing system. Each of the selected hens was X-rayed in the 35^th^, 51^st^ and 72^nd^ week of age. These time points were chosen due to the following reasons: By the 35^th^ week of age ossification of the keel bone is terminated [2] and egg production has reached its maximum. By the 51^st^ week of age laying performance has started to decrease to some extent and the prevalence of keel bone fractures seems to have reached a maximum according to several studies [4, 22]. By the 72^nd^ week of age laying performance has decreased considerably and commercial laying hens are often culled.

Three hens in the floor housing system (2 WLA and 1 G11) died before the last X-ray examination resulting in fewer hens in the 72^nd^ compared to the 35^th^ and 51^st^ week of age in these two groups.

**Fig 1:**
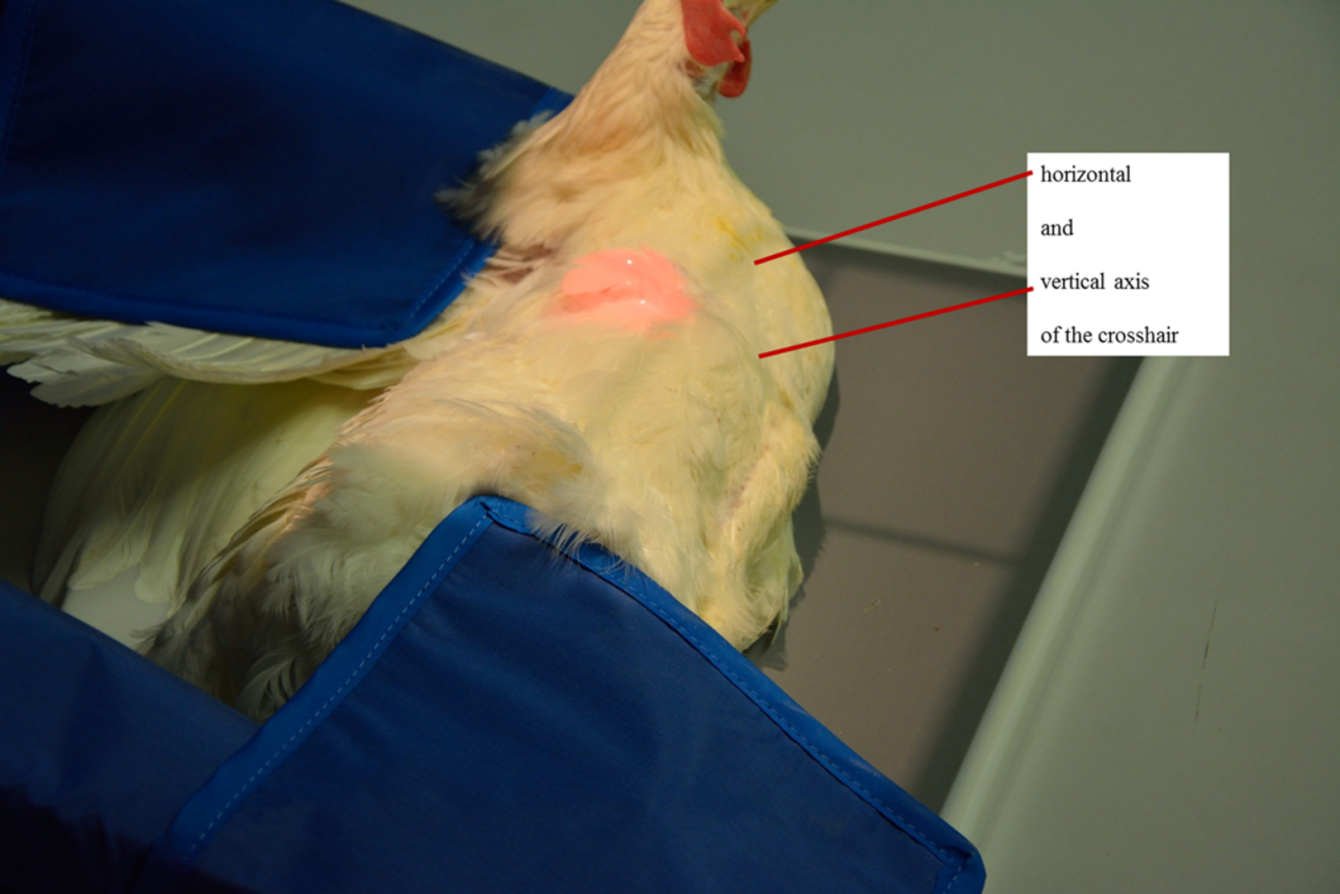
Positioning of the animal on the X-ray detector. The red lighted area indicates the radiation field center.

Digital radiographs were taken using the X-ray generator WDT Blueline 1040 HF (Wirtschaftsgenossenschaft deutscher Tierärzte eG, Garbsen, Germany) and the X-ray suitcase Leonardo DR mini (Oehm und Rehbein GmbH, Rostock, Germany). The non-anaesthetized hen was gently placed on its left side on a digital flat panel detector (Thales Pixium 2430 EZ wireless, Thales Electron Devices S.A., Vélizy-Villacoublay, France). One person pulled the legs caudally and a second one fixed the wings above the back of the animal to make sure that the hen was safely fixated and that the limbs did not overlie the keel bone. The hen was positioned so that the keel bone was plane and at right angles to the x-rays. The vertical axis of the crosshair was located right in front of the cranial keel bone edge and the horizontal axis divided the hen’s body into a ventral third and dorsal two-thirds. The radiation field center was located immediately above the crosshair center (Fig 1). Images were taken with 50.0 kV and at 2 mAs for each laying hen.

Initially, postero-anterior radiographs were taken as well. However, these X-rays could not be evaluated because the ventral surface of the keel bone is very small and because they resulted in projections in which other parts of the body such as the vertebral column inevitably overlay the keel bone. Consequently, we decided only to take lateral radiographs.

### Evaluation of X-ray images

The images were evaluated using the image processing system AxioVision 4.8 (Carl Zeiss Microscopy GmbH, Jena, Germany). The same person blindly evaluated all images. Each radiograph was evaluated for deviations and fractures. According to suggestions by Casey-Trott et al. [11] fractures and deviations were addressed as two separate, mutually exclusive, binary categories. This system allows an easy comparison across studies [11]. In addition, the size of the deviated keel bone area was also measured in order to get a value which indicates the severity of the deviation. Such approaches have also been encouraged by Casey-Trott et al. [11].

#### Deviations

In order to calculate the prevalence of deviations for each housing system, age and layer line, each radiograph was scored as 1 if a deviation was present and as 0 if no deviation was present.

**Fig 2:**
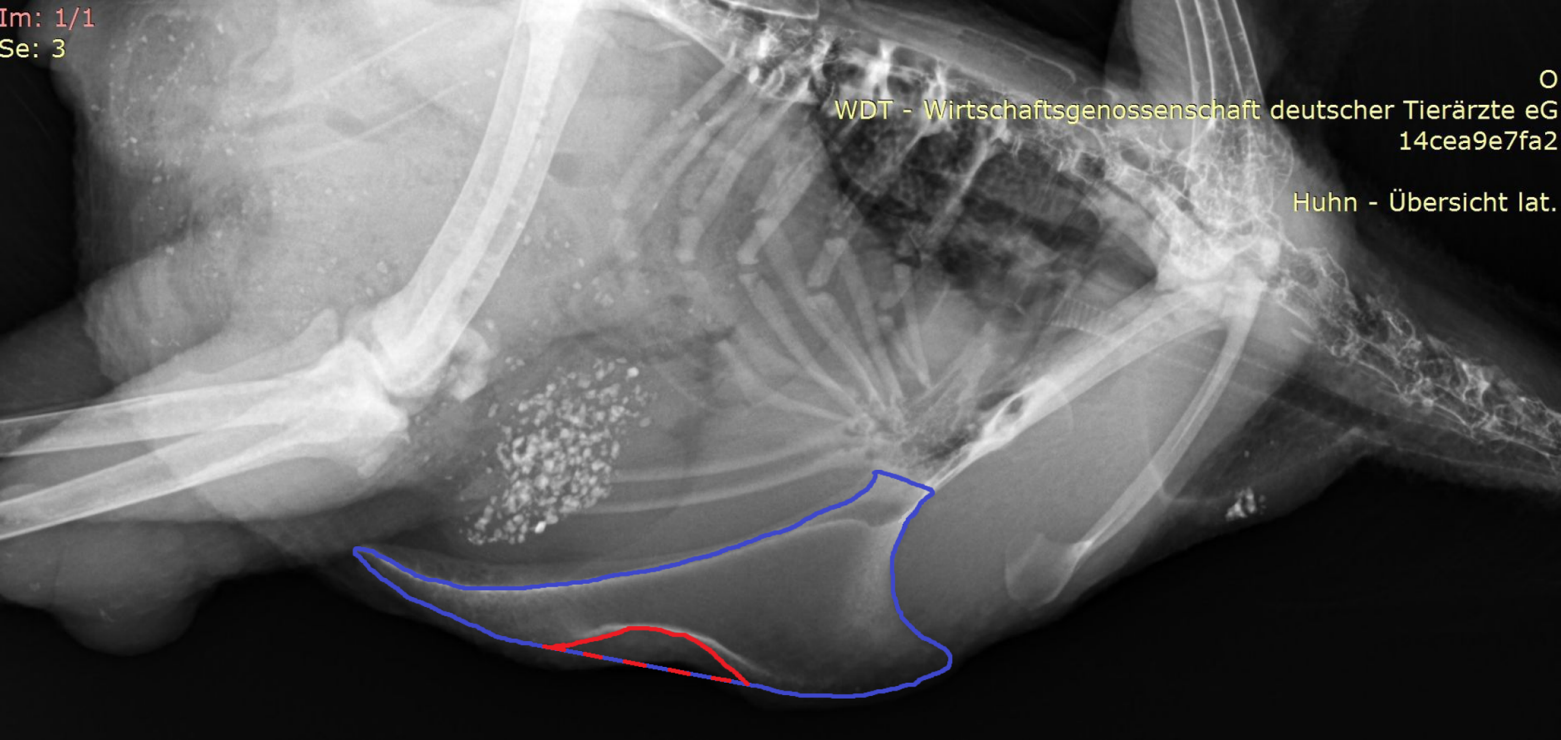
A deviated keel bone. The keel bone surface area is circumscribed with blue color; the area of deviation is circumscribed with red color. The blue-red line marks the straight line between the start and end point of thedeviated outline.

In order to estimate the severity of a deviation, the proportion of deviated keel bone area (POD) was calculated as follows: The area of deviation was estimated by circumscribing the deformed outline and linking the start and end point of this outline by a straight line (Fig 2). The size of this area was calculated by AxioVision. Afterwards, the whole keel bone was circumscribed up to the insertion of the trabecula intermedia (Fig 2) and the size of its surface area was calculated by AxioVision. Again, the start and end point of the deformed outline were linked with a straight line (Fig 2) as an estimate for the size of the actual keel bone surface area. Finally, POD was calculated by dividing the area of deviation by the keel bone surface area.

#### Fractures

**Fig 3:**
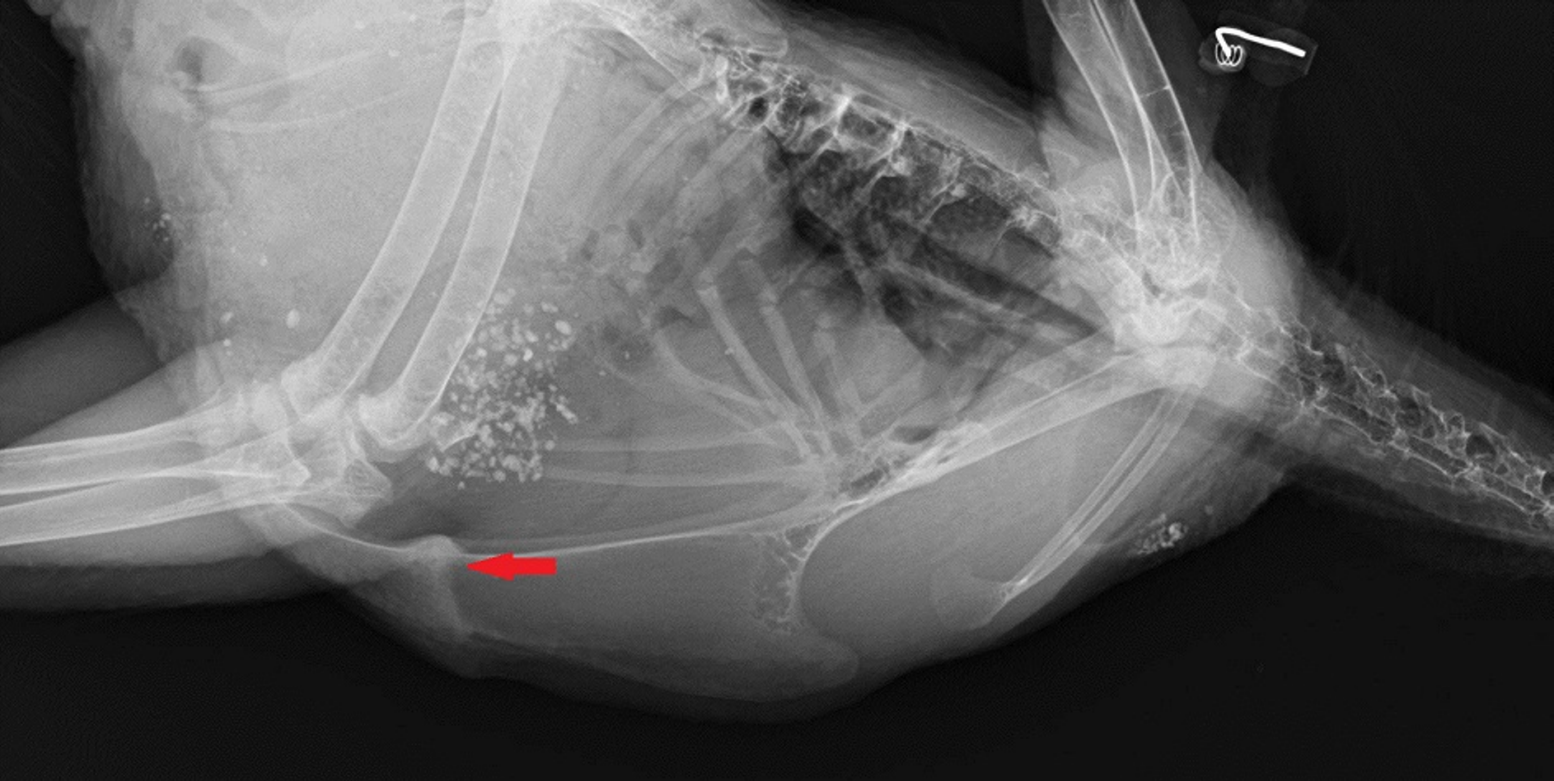
A fractured keel bone with callus formation. The arrow shows the fracture callus.

**Fig 4:**
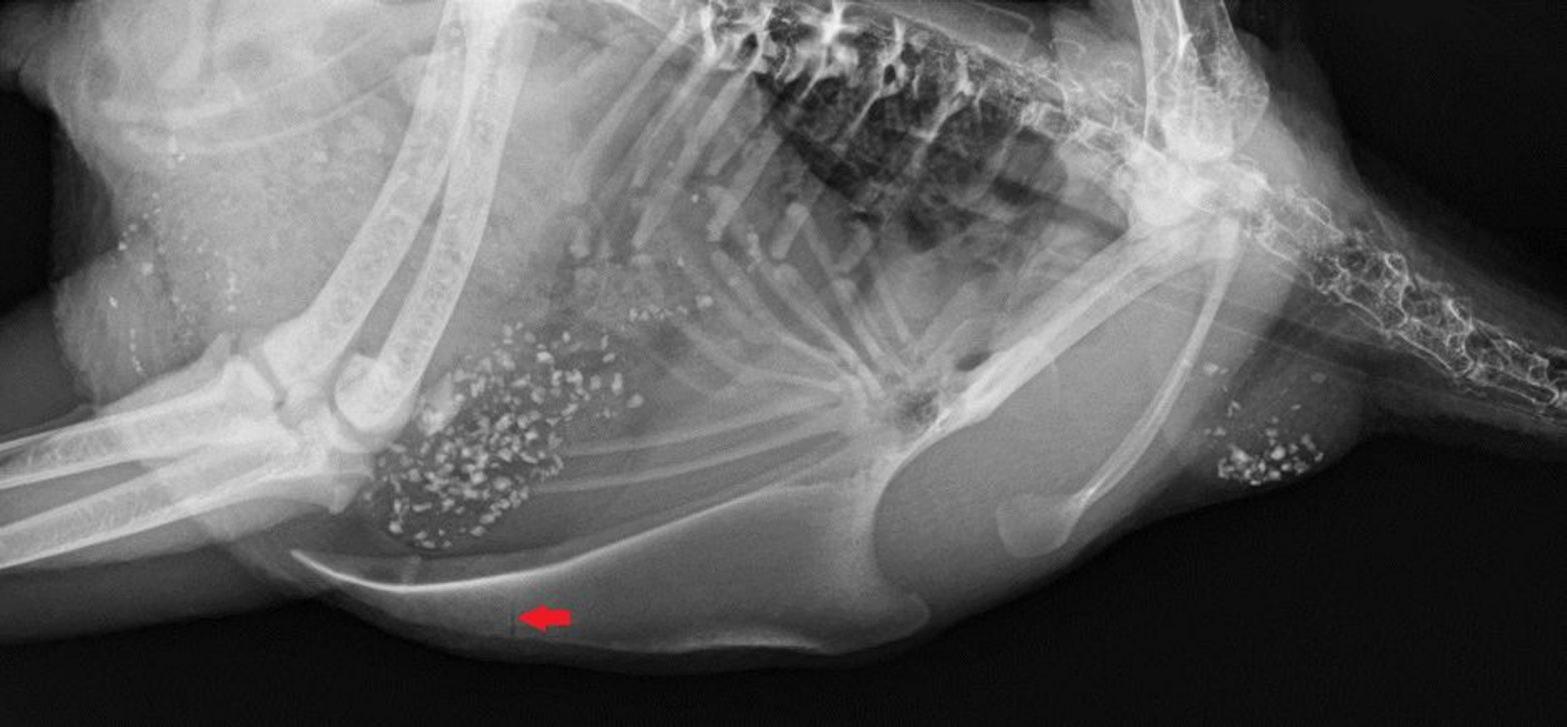
A fractured keel bone without callus formation. The arrow shows the fracture, a thin, black line.

Fractures were defined as sections of the keel bone with thickened bone (callus) in the image (Fig 3) or as black, thin lines, indicating a fracture without callus formation (Fig 4). In order to calculate the fracture prevalence for each housing system, age and layer line, each radiograph was scored as 1 if any type of one or several fractures was present and as 0 if no fractures were present.

### Statistical analysis

Data were analyzed using the statistical software packages JMP 11 (SAS Institute Inc., 2014) and R 3.3.3 (R Core Team, 2017). For statistical analysis of the prevalence of deviations, hens with a deviated keel bone were scored as 1 and those without any deviation as 0 at each time point. The effects of housing system (cages and floor housing system), layer line (BLA, L68, WLA, R11 and G11) and age (35^th^, 51^st^ and 72^nd^ week of age) on the binary outcome variable “deviation” were analyzed by means of Chi-square test and Fisher’s exact test, respectively. For housing system and layer line a separate Chi-square or Fisher’s exact test was performed for each of the three weeks of age. Data are presented as total numbers of affected animals. Hens without keel bone deviation were excluded from analysis of POD. A multifactorial ANOVA was applied to log-transformed POD. The mixed model contained the fixed effects housing system, layer line, age as a categorical variable and all interactions. To consider the repeated measures, animal was included as random effect. Multiple comparisons of means were performed using the Tukey’s HSD test. Data for POD are presented as back-transformed LSM and upper and lower bounds of the 95% confidence interval. For statistical analysis of fractures, birds with one or multiple fractures were scored as 1 and those without any fracture as 0 at each time point. The effects of housing system (cages and floor housing system), layer line (BLA, L68, WLA, R11 and G11) and age (35^th^, 51^st^ and 72^nd^ week of age) on the binary outcome variable “fracture” were analyzed by means of Chi-square and Fisher’s exact test, respectively. For housing system and layer line a separate Chi-square or Fisher’s exact test was performed for each of the three weeks of age. If it was not possible to evaluate the keel bone for fractures or deviations because the legs of the hen overlay the keel bone in the radiograph, this image was excluded from analysis. The number of hens included in the analysis of prevalence of deviations and fractures is indicated in each figure.

## Results

### Deviations

**Fig 5:**
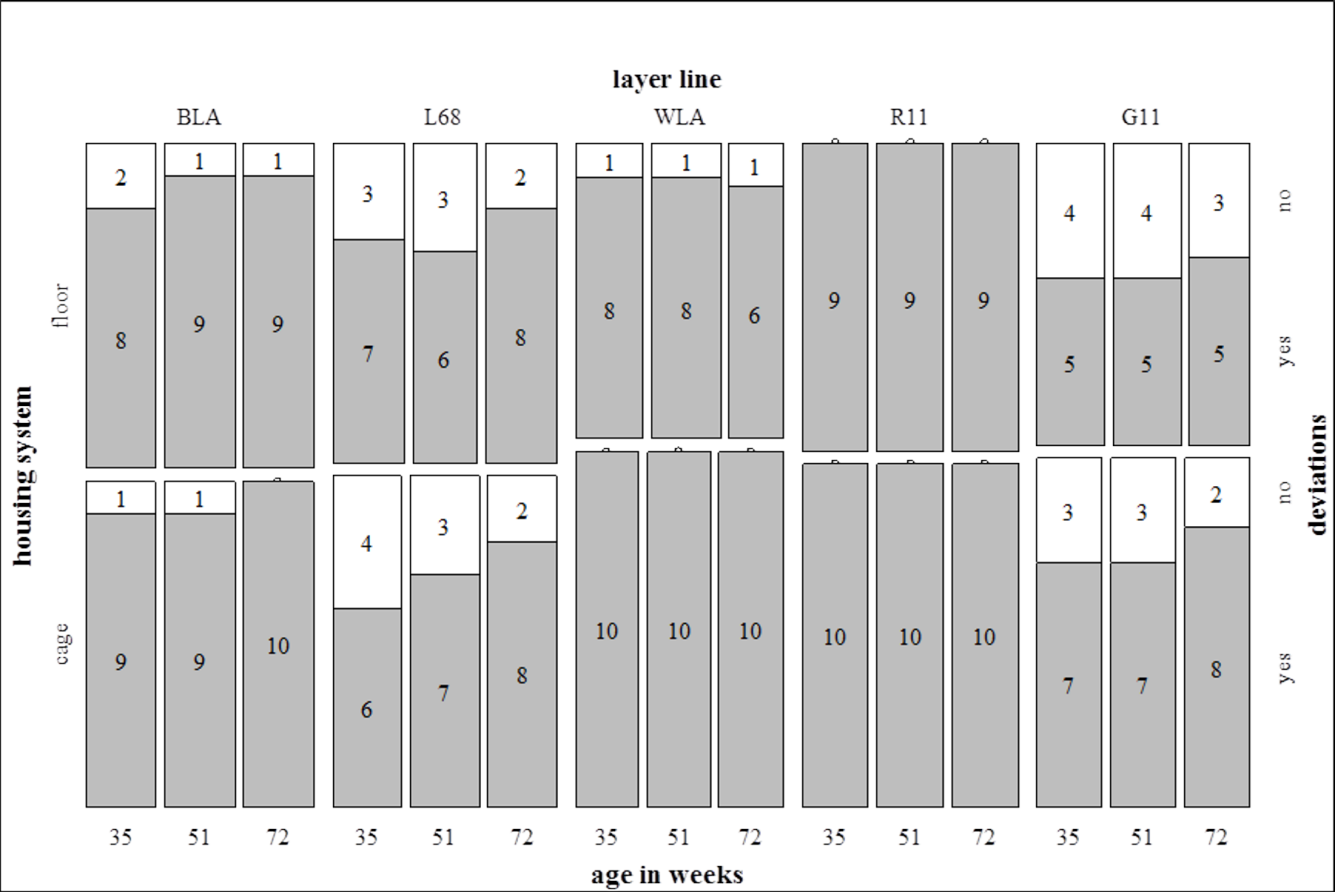
Prevalence of deviations. Each bar represents the total amount of radiographed and evaluated hens of one layer line within one housing system. The grey part of a bar shows the part of hens with a deviated keel bone, the white part those without any deviation. The numbers written in the bars show the total numbers of affected or non-affected hens, respectively. Prevalence of deviations was affected by layer line (p<0.05). There was no significant influence of housing system (p>0.05) or age (p>0.05) on prevalence of deviations.

The prevalence of keel bone deviations did not differ significantly between housing systems or weeks of age but between layer lines (p<0.05 for each age) (Fig 5). The highest prevalence was found in R11: all animals of this layer line had a deviated keel bone at all ages. The lowest prevalence of deviations was found in G11 (35^th^, 51^st^ and 72^nd^ week of age: 63.2%, 63.2% and 72.2%), followed by L68 (65%, 68.4% and 80%), BLA (85%, 95% and 95%) and WLA (94.7%, 94.7% and 94.1%).

**Fig 6:**
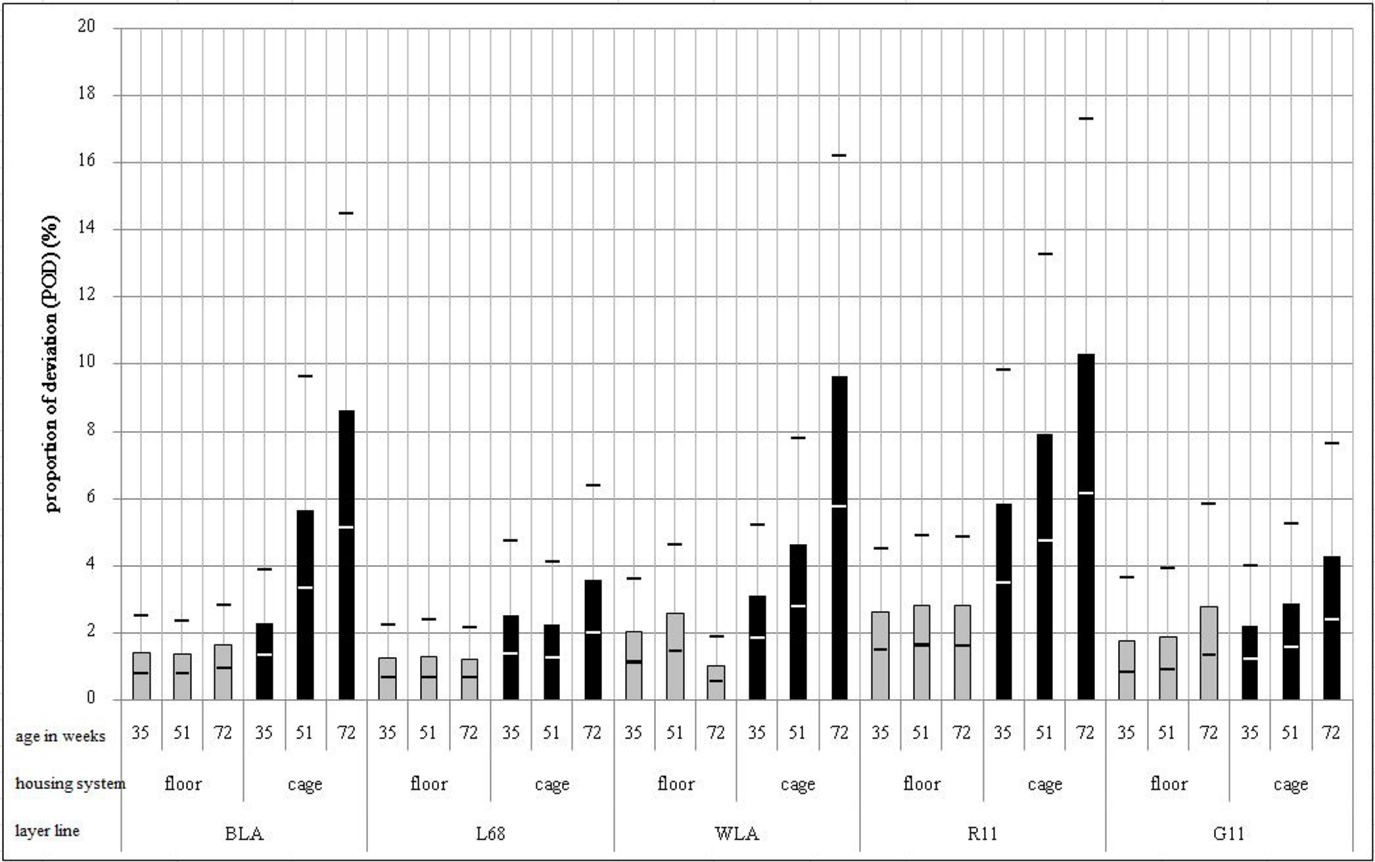
Proportion of deviated keel bone area (POD) (back-transformed LSM and upper and lower bounds of the 95% confidence interval) in %. POD was affected by the three-way interaction between housing system, layer line and age (p<0.01).

The severity of deviations, measured as POD, was significantly affected by the three-way interaction between housing system, layer line and age (p<0.01) (Fig 6). In the floor housing system POD did not differ significantly between weeks of age in any of the five layer lines (p>0.05). In the cage system, in contrast, POD was significantly higher in the 72^nd^ compared to the 35^th^ week of age in BLA (back-transformed LSM and 95% confidence interval: 35^th^ week of age: 1.34% < 2.28% < 3.88%; 72^nd^ week of age: 5.14% < 8.63% < 14.47%; p<0.05), WLA (35^th^ week of age: 1.84% < 3.09% < 5.19%; 72^nd^ week of age: 5.75% < 9.65% < 16.19%; p<0.05) and R11 (35^th^ week of age: 3.49% < 5.86% < 9.83%; 72^nd^ week of age: 6.14% < 10.30% < 17.29%; p<0.05). This was not the case in L68 and G11 (p>0.05). POD was higher in cages compared to the floor housing system in the 72^nd^ week of age in WLA and R11 (WLA floor housing: 0.55% < 1.02% < 1.89%; R11 floor housing: 1.63% < 2.81% < 4.85%; WLA and R11 in cages see above; p<0.05). In BLA, L68 and G11 POD did not differ significantly between housing systems in any week of age (p>0.05).

### Fractures

**Fig 7:**
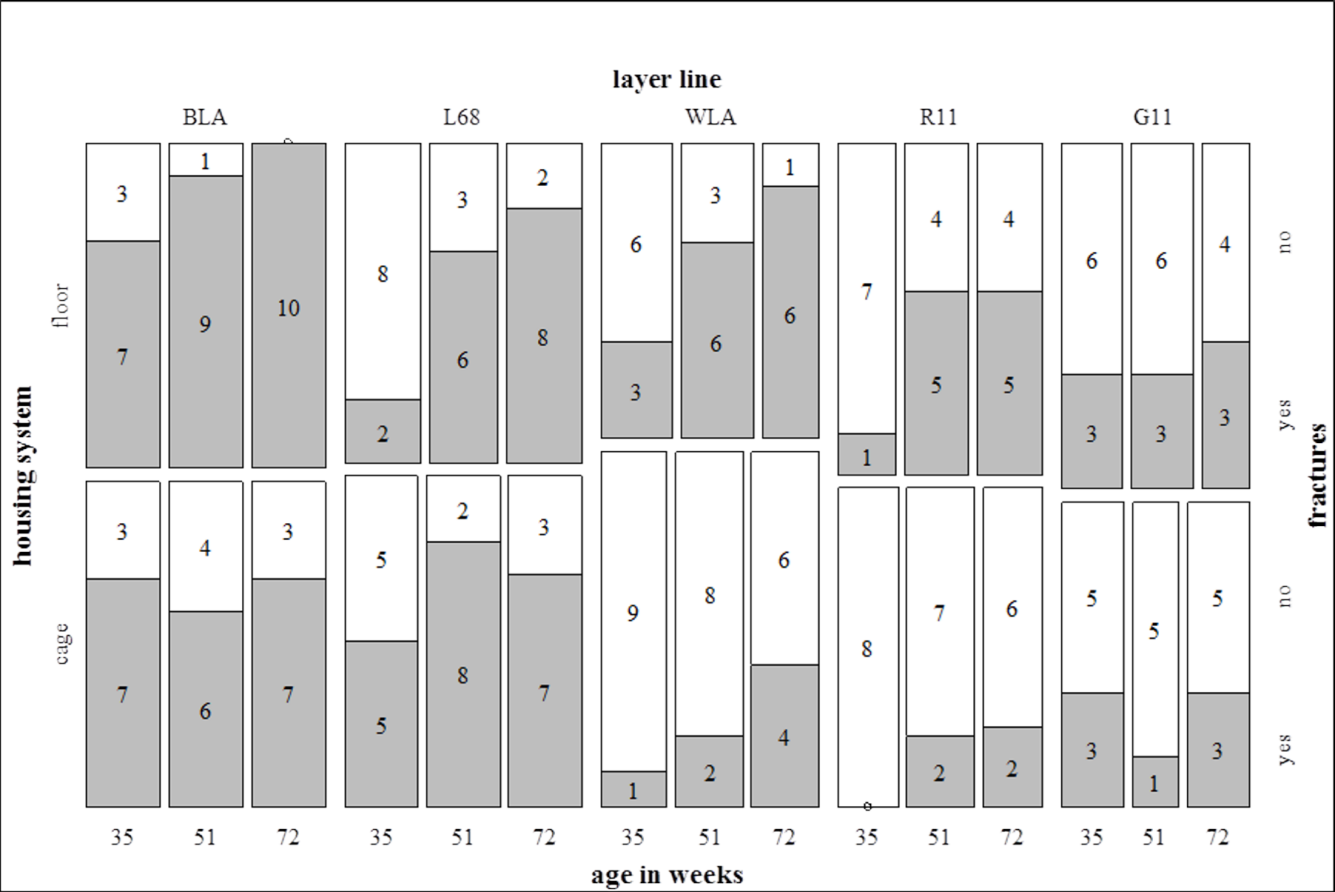
Fracture prevalence. Each bar represents the total amount of radiographed and evaluated hens of one layer line within one housing system. The grey part of a bar shows the part of hens with a fractured keel bone, the white part those without any fracture. The numbers written in the bars show the total numbers of affected or non-affected hens, respectively. Fracture prevalence was affected by layer line (p<0.05 at all three ages) and age (p<0.001). Housing system significantly influenced fracture prevalence in the 72^nd^ week of age (p<0.05).

Fracture prevalence increased with age (35^th^, 51^st^ and 72^nd^ week of age: 34.8%, 52.7% and 62.2% of all hens affected; p<0.001) (Fig 7).

There was no significant difference between housing systems in the 35^th^ (floor housing: 34.8%, cages: 34.8% affected hens; p>0.05) and 51^st^ week of age (floor housing: 61.7%, cages: 44.2% affected hens; p>0.05), but in the 72^nd^ week of age fracture prevalence was significantly higher in the floor housing system compared to cages (floor housing: 73.3%, cages: 50%; p<0.05) (Fig 7).

Moreover, fracture prevalence differed significantly between layer lines at all three ages (p<0.05) (Fig 7). BLA and L68 showed a higher prevalence of fractures than WLA, R11 and G11 in the 51^st^ and 72^nd^ week of age (percentage of affected animals in the 35^th^, 51^st^ and 72^nd^ week of age: BLA: 70%, 78.9% and 85%; L68: 35%, 73.7% and 75%; WLA: 21%, 44.4% and 61.1%; R11: 6.3%, 38.9% and 41.2%; G11: 35.3%, 25% and 37.5%). Within the brown layer lines there were significantly more hens with fractures in the high performing line BLA compared to the low performing line L68 (p<0.05). Within the white layer lines there was no significant difference between high and low performing lines (p>0.05).

## Discussion

The current experimental study confirmed an alarmingly high prevalence of keel bone fractures and deviations in laying hens.

34.8% of all radiographed laying hens had a keel bone fracture in the 35^th^ week of age. In the 51^st^ week of age the prevalence was 52.7% and in the 72^nd^ week of age 62.2%. These numbers are comparable to findings by Petrik et al. [4] who found an overall keel bone fracture prevalence of 36% at 35 and of 46.3% at 50 weeks of age in nine farms with conventional cages and eight farms with floor-housed flocks in Ontario, Canada. In other studies, fracture prevalence was even higher. Heerkens et al. [5] found an overall fracture prevalence of 60% at 29, 76% at 39 and 86.5% at 49 weeks of age in an experimental setup with 96 palpated hens that were housed in pens either with or without ramps. In a field study with 67 flocks in the UK, assigned to eight subcategories of housing system, Wilkins et al. [7] found 36% affected hens in furnished cages and 86% affected hens in a free-range system with aerial perches in the indoor house at the end of the production period.

The prevalence of deviations in the current study was 81% in the 35^th^, 83% in the 51^st^ and 88% in the 72^nd^ week of age and was higher compared to other studies. In the study by Heerkens et al. [5], which has been mentioned above, 31.2% of keels were found to be deviated with 39 weeks of age and 49% with 49 weeks of age via palpation. Käppeli et al. [21] reported a prevalence of 46.7% with 37, 64.6% with 43 and 72.9% with 62 weeks of age in one experiment in which 240 floor-housed hens were palpated at each time point. The higher prevalence of deviations in the current study might be explained by the fact that we used radiography to detect deviations which is more sensitive than palpation.

Due to the high prevalence and the probable painfulness of keel bone fractures [9, 10] it is crucial to investigate this multifactorial animal welfare problem into more detail.

### Radiography as a tool to assess KBD throughout the laying period

In this study, we evaluated keel bone fractures and deviations throughout the laying period using radiography. For longitudinal studies which have investigated KBD at different ages, most research groups have used palpation [4, 5, 21, 22, 28]. This is an appropriate method, especially for a large number of animals and on-farm assessment, as it is quick, well-validated and cost-efficient [11, 38]. However, not all fractures, especially mild fractures and those at the dorsal aspect of the keel bone, can be detected by palpation [34]. Radiography has been shown to give a more detailed insight into the bone [34–36, 39]. Clark et al. [35] used radiography to detect fractures in the skeleton of laying hens, including the keel bone. They also detected and measured indentations of the keel bone. For their study, the hens had been euthanized before radiographs were taken. Richards et al. [34] took radiographs of live, sedated hens to detect keel bone fractures. The duration of the study was six weeks because the focus was on the healing process and not on the fracture prevalence at different ages. Our study is therefore the first published work in which radiographs have been used to assess keel bone fractures and deviations throughout the laying period. It was possible to get radiographs with a high resolution which showed the whole keel bone. Also small fractures without callus formation could be detected which might not have been the case with palpation. Furthermore, a clear differentiation between fractures and deviations was possible. Moreover, we presented a new method to measure keel bone deviations in relation to the whole keel bone. With this method we were able to measure the severity of deviations. Thus, no scoring system was necessary. Compared to a scoring system, calculating the proportion of deviated area allows assessing deviations objectively on a continuous scale. This increases the sensitivity of the measure and allows comparing keel bones with even very slight differences. Due to varying keel bone shape, it is not possible to accurately infer the intact outline of a keel bone once it is deviated. Consequently, we decided to draw a straight line between the start and end point of the deviated outline to get the most accurate estimation of the size of the deviated area. Even though this approach might underestimate the size of the deviated area, in our opinion this was the most accurate, objective and reproducible method to estimate the size of the deviated area.

In the current study the hens were not sedated for radiography. This required that two persons had to handle each hen. With our handling, all laying hens were immobilized and we did not have problems with hens moving. However, the safety of the two persons handling the animal is also an important factor. We measured the radiation dosage which was received by the two persons with dosimeters at the fingers and at the torso. The dosage was far below the maximal permitted dosage (data not shown) which was, most probable, because of the modern X-ray generator which does not emit a lot of radiation outside the radiation field. However, to avoid that persons have to be close to the generator, another method would be required. Sirovnik and Toscano [36] conducted a study on radiography in which they hung the hens upside down so that they remained still without further fixation. This seems to be a promising alternative to the handling of the animals.

In conclusion, radiography has been shown to be a good tool to assess keel bone fractures and deviations throughout the laying period. As the equipment which we used to take radiographs is portable, the presented method can also be used for on-farm assessment. It is, however, more expensive and more time-consuming compared to palpation which might therefore be more suitable for large-scale studies.

### Housing system

Our hypothesis that laying hens in cages would show more keel bone deviations compared to hens in the floor housing system was not confirmed since prevalence of deviations did not differ between the systems at any age. However, as predicted, keel bone deviations were more severe in cages in the two layer lines WLA and R11 as can be seen by the fact that POD was larger compared to the floor housing system in the 72^nd^ week of age in these two layer lines. Severity of deviations, i.e. POD, also increased with age in the cage system in the layer lines BLA, WLA and R11, but not in the floor housing system. Keutgen et al. [17] found a higher prevalence of deviations in free-range and deep litter stocks than in conventional cages. However, in our study cages were enriched with a perch which might explain the different findings because of the pressure on the keel bone caused by a perch. The impact of perches on the keel bone has been shown in different studies. Pickel et al. [23] showed that in laying hens sitting on a perch, the peak force induced by the perch was approximately 5 times higher on the keel bone compared to the peak force on a single foot pad. This indicates that hens have most of their weight on the keel bone during perching. Moreover, Stratmann et al. [22] showed that prevalence of deviations could be lowered by covering the perches with a soft material.

The present results of lower POD in the 72^nd^ week of age in floor-housed compared to caged hens in some layer lines confirm the importance of movement for bone strength that has been shown by several authors [24, 26, 27]. The lack of movement, combined with the constant pressure on the keel bone caused by the perch, might have led to the high and increasing POD in caged laying hens.

As predicted, fracture prevalence was significantly higher for the laying hens in the floor housing system compared to the hens in cages in the 72^nd^ week of age. The higher fracture prevalence in floor housing corresponds to findings of other studies [4, 7, 40]. These findings can be explained by the higher risk of collisions with perches, nests and other hens that birds in floor housing systems are exposed to compared to birds in single cages [7, 18]. The fact that fracture prevalence did not differ significantly between the housing systems in the 35^th^ and 51^st^ week of age might be explained by the general increase in fracture prevalence which was more marked for the hens kept in the floor housing system. Fracture prevalence was also high in cages, although these hens were at a lower risk of collisions. We assume that the fractures in cages were rather pathologic fractures due to bone weakness than of traumatic origin. In order to confirm this assumption, a histological examination of the keel bones would be required.

Our results concerning the differences between the two housing systems show that there seem to be different risks and causes for deviations on one hand and fractures on the other hand: Deviations are more severe in cages possibly due to lack of movement and a resulting deterioration of bone strength [24, 26, 27], combined with the constant pressure on the keel bone. Fractures are more common in the floor housing system probably due to a higher risk of accidents and collisions [7, 18].

### Genetic background

We found significant differences between the five layer lines which differed in phylogenetic background (brown versus white layer lines) and in laying performance (high versus low performing lines).

In contrast to expectations and other studies [19, 28], we did not find more deviations in brown compared to white layer lines. In terms of prevalence of deviations there were differences between the layer lines but these could not clearly be classified by “white” versus “brown”. Within the hens affected by keel bone deviations, POD tended to be higher in white layer lines. As body weight was lower in white layer lines which tended to show a higher POD and which, in total, did not show a lower prevalence of deviations compared to brown layer lines, we cannot confirm the assumption made by other authors that the prevalence and severity of keel bone deviations increase with body weight [19, 28].

Taken together, prevalence of deviations and POD might indicate that keel bone strength was higher in brown compared to white layer lines. This assumption is in accordance with other studies that showed that breaking strength of at least some of the long bones is higher in brown compared to white hybrids [19, 41]. Vits et al. found a higher humerus breaking strength in LB compared to LSL hens. No significant difference was found in tibia breaking strength [19]. Riczu et al. found a higher humerus and femur breaking strength in the brown-egg strain Shaver 579 compared to the white-egg strain Shaver 2000 [41].

As hypothesized, brown layer lines showed significantly more keel bone fractures than white layer lines in the 51^st^ and 72^nd^ week of age. This finding is consistent with a study by Heerkens et al. [5] who found a higher fracture prevalence in ISA Brown compared to Dekalb White hens. The higher fracture prevalence in brown layers might be explained by differences between white and brown layers with respect to motor skills. White layers have been found to show better flight and 3D-movement skills and, thus, their risk for collisions with housing equipment such as perches might be lower compared to brown layers [5, 42]. Another suggested explanation is that, as brown layers usually have a higher body weight, the impact of collisions could be higher [5, 22]. Both factors are likely to lead to higher bone fracture prevalence in brown than in white layers. Candelotto et al. [30] found fewer experimental fractures in two brown layer lines compared to other lines, including white layer lines. This is in contrast to our findings. However, they investigated the susceptibility to fractures in recently euthanized hens using an impact test apparatus. Thus, behavioral confounds were eliminated. Their results therefore give a further hint that higher fracture prevalence in the brown layer lines in our study was probably mainly due to differences in flight abilities as mentioned above and not due to non-behavioral factors like bone strength.

Our hypothesis that high performing layer lines would show more keel bone damage could neither be rejected nor supported. On one hand, findings within the brown layer lines confirmed this hypothesis because the low performing line L68 had fewer deviations and fractures and the severity of deviations increased with age for the high performing line BLA but not for L68. On the other hand, within the white layer lines there was no clear difference between the high performing line WLA and both low performing lines R11 and G11. Whereas G11 had a better keel bone health than WLA, this was not the case for R11. This shows that other differences between the white layer lines might have played a more important role than the difference in laying performance. Possible differences might be endocrine or behavioral factors.

In a comparative study of commercial breeds with a high laying performance and traditional breeds with a significantly lower laying performance Hocking et al. [33] found differences of bone parameters between the breeds. Traditional breeds had a higher breaking strength of humeri and tibiotarsi and also a higher radiographic density of keel bones and tibiotarsi compared to commercial breeds. The results of that study and of our study together indicate that selection on high productivity may have led to poor bone quality and that further research should be done in this field.

Altogether, the observed differences regarding genetic background confirm that the incidence of keel bone damage is influenced by layer line. However, susceptibility to deviations on one hand and fractures on the other hand do not seem to be directly linked.

### Age

In contrast to expectations and to findings by Habig et al. [28], age did not have any significant effect on prevalence of keel bone deviations and POD only increased for some layer lines in cages.

As predicted, fracture prevalence increased with age. This corresponds to other studies [26, 28] and is consistent with findings that bone strength deteriorates with age [43]. However, some studies showed an increase of fractures only until a certain age of about 50 weeks [4, 22]. This was not confirmed in the present study where a higher fracture prevalence was found in the 72^nd^ compared to the 51^st^ week of age.

## Conclusions

This experimental study has confirmed the relevance of keel bone damage in laying hens and the influence of the housing system and phylogenetic background on prevalence of KBD. Furthermore, indicators that selection on high performance might influence the prevalence of KBD have been found. Lastly, we have presented a new method to evaluate keel bone fractures and deviations throughout the life of a laying hen with radiography.

## Supporting information

S1 Data Complete data set. (XLSX)
